# Medical management of gastric cancer: a 2017 update

**DOI:** 10.1002/cam4.1274

**Published:** 2017-12-13

**Authors:** Nikolaos Charalampakis, Panagiota Economopoulou, Ioannis Kotsantis, Maria Tolia, Dimitrios Schizas, Theodore Liakakos, Elena Elimova, Jaffer A. Ajani, Amanda Psyrri

**Affiliations:** ^1^ Department of Internal Medicine Section of Medical Oncology Attikon University Hospital National and Kapodistrian University of Athens School of Medicine Athens Greece; ^2^ Radiation Oncology Department University of Thessaly School of Health Sciences Faculty of Medicine Larissa Greece; ^3^ First Department of Surgery National and Kapodistrian University of Athens School of Medicine Athens Greece; ^4^ Department of Medicine Division of Medical Oncology Princess Margaret Cancer Centre University of Toronto Toronto Ontario Canada; ^5^ Department of Gastrointestinal Medical Oncology The University of Texas MD Anderson Cancer Center Houston Texas

**Keywords:** Gastric cancer, molecular characteristics, multimodality treatment approach, new therapies, pathologic characteristics

## Abstract

Gastric cancer remains a considerable health burden throughout the world. The Cancer Genome Atlas (TCGA) analysis has recently unveiled 4 genotypes of gastric cancer with data not ready to change treatment strategy yet. A multimodality approach to therapy is the cornerstone of screening, diagnosing, staging, treating and supporting patients with gastric cancer. The evidence‐based approach to localized gastric cancer (>cT1b) is to use an either preoperative or postoperative strategy to maximize the benefit of surgery. The focus of future research is to optimize chemotherapy regimens, determine the role of radiation therapy and investigate the effect of treatment timing. In metastatic gastric cancer, biologic therapies have been introduced targeting markers shown to be prognostic. The results of ongoing randomized controlled phase 3 trials using targeted and immunotherapy agents, either in combination or alone, have the potential to alter the current treatment landscape of advanced gastric cancer.

## Introduction

Gastric cancer represents the fifth most common malignancy and despite a steady decline remains the third leading cause of cancer mortality with widely varying incidence worldwide. The highest incidence (>20 per 100,000 in men) is seen in China, Japan, Latin America, and Eastern Europe, whereas the lowest incidence (<10 per 100,000 in men) is seen in North America, parts of Africa and Northern Europe 1. Only 27% of newly diagnosed gastric cancers are localized with a 5‐year overall survival (OS) rate of 30.4%, which remains stable over the last 30–40 years 2. Surgery is still the only chance for cure and implementation of a multimodality treatment approach is utilized to further improve survival. Advanced disease carries a dismal prognosis and treatment remains challenging with a 5‐year OS rate <5%. Thus despite decreasing incidence, gastric cancer remains a serious health burden globally with high mortality rate.

## Etiologic Characteristics and Risk Factors

Two different mechanisms of carcinogenesis have been proposed, correlating with two histologic variants, diffuse and intestinal. Intestinal‐type gastric adenocarcinoma likely begins with Helicobacter pylori infection that leads to multistep progression 3. A new joint report from the World Cancer Research Fund and the American Institute for Cancer Research looking at causes of gastric cancer found three new somewhat surprising correlating factors: alcohol, processed meat, and obesity 4. While all three are associated with several other cancers including colon cancer and breast cancer, this is the first time they have been associated with gastric cancer. Besides, diffuse‐type gastric adenocarcinoma results from defective intracellular adhesion molecules due to loss of E‐cadherin protein expression that is encoded by *Cadherin 1 (CDH1)* gene 5. Despite recent progress, the precise etiologic features of gastric cancer and the relationship between the environment and the host are unknown. Further research is warranted with the view to elucidate the tumorigenesis of gastric cancer.

## Pathologic and Molecular Characteristics

Analysis from the Cancer Genome Atlas (TCGA) project has recently uncovered four distinct genotypes of gastric cancer 6. While it is not clear if these genotypes will ultimately guide patient therapy, the four following major genomic subtypes of gastric cancer with histological and etiological heterogeneity have been identified:

1. Tumors containing Epstein–Barr virus (EBV), where high prevalence of DNA hypermethylation, amplification of *JAK2* and of known suppressors of immune response programmed death ligands 1 (*PD‐L1)* and 2 (*PD‐L2)* genes are common. This group accounts for approximately 10% of the cancers, with nearly 80% having a protein‐changing alteration in the *PIK3CA* gene pathway.
2. Tumors showing microsatellite instability, where a high rate of mutations, including mutations of genes encoding targetable oncogenic signaling proteins take place due to malfunctioning in the DNA repair mechanisms. Approximately 20% of tumors fall into this group.
3. The majority of tumors are categorized as “chromosomally unstable.” These tumors display marked aneuploidy and have a considerable number of genomic amplifications of key receptor tyrosine kinases, cell cycle regulation genes and transcription factors. This group represents approximately half of the cancer specimens (50%) and is frequently found in the gastroesophageal junction (GEJ).
4. The last group is classified as “genomically stable”, lacks the molecular characteristics of the other three subtypes and has tumors enriched for the diffuse histologic variant, with approximately 30% of them having mutations or fusions in the *RHOA* signaling pathway. This group accounts for 20% of gastric cancers that are characterized by the lack of high levels of aneuploidy and high metastatic potential.

This classification may be used supplementary to histopathology to provide patient stratification as a guide to targeted agents. The TCGA genotypes have now been validated as prognostic 7. Additionally, the Asian Cancer Research Group (ACRG) classified gastric cancer into four subtypes based on gene expression data and made a correlation with postsurgical relapse patterns and survival outcomes 8. The worst prognosis stands for mesenchymal‐like tumors, followed by TP53‐inactive, TP53‐active, and the best for microsatellite‐instability tumors. The TCGA and ACRG classification systems share similarities but also have differences implying that they are related but distinct.

## Treatment

### Resectable disease

#### Surgery

Surgical resection (R0) remains the only curative modality for localized gastric cancer. However, survival is poor (20–50% at 5 years) with surgery alone, necessitating efforts to improve the outcomes for this group of patients using perioperative chemotherapy 9 or postoperative (adjuvant) chemoradiotherapy 10.

Surgeons in Japan carry out extended lymphadenectomy as routine practice, whereas in the United States, 54% of primary gastrectomy patients undergo less than a D1 lymphadenectomy 10. A D1 lymphadenectomy is defined by removal of the perigastric lymph nodes, and D2 by the extended dissection of nodes along the left gastric, celiac, hepatic, and splenic arteries, as well as those in the splenic hilum. Despite some disagreement about the benefits of D2 dissection, most experts agree that localized gastric cancer with clinical stage >T1b is best treated with multidisciplinary approaches and particularly within high volume centers 11. However, efforts to identify reliable criteria in order to properly select patients for multimodal treatment are urgently needed. Some pretherapeutic tumor features, such as tumor site, grading, Lauren's histologic subtype and the presence of signet‐ring cells have been associated with grade of response, trying not to underestimate the importance of an adequate surgery. This issue has not been solved yet and represents a challenge to be addressed in the future 12.

In this review, we are trying to address multimodality approach exploring the effect of treatment timing (preoperative, postoperative, or both). Our criteria for selection of cited studies are to include the major phase 3 studies and analyze the more recent trials incorporating targeted and immunotherapy agents, ignoring older and negative trials.

#### Perioperative chemotherapy

The MAGIC trial has established Level 1 evidence for the perioperative approach 9. A total of 503 patients with gastric, GEJ and esophageal adenocarcinoma were enrolled and then randomized to either perioperative chemotherapy consisting of epirubicin, cisplatin, and infusional 5‐fluorouracil (5‐FU) (ECF) and surgery or surgery alone. Postoperative chemotherapy was associated with toxicity. In this trial, only 34% of patients received this treatment and only 68% of patients proceeded with surgery. ECF, though, improved both progression‐free survival (PFS) and OS [Hazard Ratio (HR) for progression = 0.66, 95% CI: 0.53–0.81, *P* < 0.001 and HR for death = 0.75, 95% CI: 0.60–0.93, *P* = 0.009].

However, the addition of perioperative chemotherapy did not show any benefit in the EORTC 40954 study by the European Organization for Research and Treatment of Cancer 13. The lack of a survival advantage, except for the fact that the trial was not powered enough, could be also attributed to the much higher rates of D2 resection (over 92% in both arms in contrast to 43% in the MAGIC trial), which could mitigate the benefit of preoperative chemotherapy 14. Thus, the main evidence for the efficacy of preoperative chemotherapy results from a series of patients who mainly had a ‘‘suboptimal’’ lymphadenectomy leading to the hypothesis that preoperative chemotherapy could fill the survival gap of a limited surgery 12.

On the other hand, an ongoing Japanese trial (JCOG0501) compares their standard of care which is surgery followed by adjuvant S‐1 chemotherapy, with neoadjuvant cisplatin and S‐1 (an oral fluoropyrimidine) 15. Although results of this trial are highly awaited, different tumor biology in the Japanese population makes it questionable whether they will be implemented in the Western population. After reviewing neoadjuvant therapy trials, we should at this point underline the significant bias related to patient selection according to tumor site (esophagus, GEJ and/or stomach; indicatively, in MAGIC trial, about 15% of the tumors located in the lower esophagus and 11% in the GEJ).

At ASCO this year, the multicenter randomized FLOT4‐AIO phase 3 trial compared perioperative chemotherapy with the taxane‐based triplet FLOT (docetaxel, oxaliplatin, and 5‐FU/LV) to the anthracycline‐based triplet ECF/ECX for 716 patients with localized gastric or GEJ adenocarcinoma 16. 37% of patients with ECF/ECX versus 50% with FLOT completed planned perioperative chemotherapy. FLOT improved median OS (35 months with ECF/ECX versus 50 months with FLOT; HR = 0.77, 95% CI: 0.63–0.94, *P* = 0.012). FLOT also enhanced median PFS (18 months with ECF/ECX versus 30 months with FLOT; HR = 0.75, 95% CI: 0.62–0.91, *P* = 0.004). However, many censored patients are still being followed. After 24 months, the difference in curves is made up by only few patients; therefore, there is need for further follow‐up. FLOT is quite toxic with up to 7% 90‐day mortality in potentially curable patients. There is also high rate of comorbidities and it is not a good platform for drug development. We suspect that FLOT is unlikely to be used in Asia and will have patchy uptake in rest of the world. This regimen should thus be used with extreme caution.

#### Postoperative chemoradiotherapy

The indication of adjuvant chemoradiotherapy comes from the Intergroup‐0116 trial (“Macdonald regimen”) for completely resected high‐risk gastric or GEJ cancer which demonstrated a significant OS benefit in favor of adjuvant chemoradiotherapy 10. This benefit in terms of OS and recurrence‐free survival (RFS) is persistent after a more than 10 years median follow‐up 17. The main limitation of this study was the inadequate lymph node dissection in the majority of the patients. A suboptimal D0 resection was performed in more than half of the patients and only 10% underwent a D2 nodal dissection questioning whether this OS and RFS benefit was a true benefit of chemoradiotherapy or this was a result of inadequate surgery.

The ARTIST trial compared the benefit of adjuvant chemoradiotherapy versus adjuvant chemotherapy in 458 patients after an R0 resection (D2 dissection was a prerequisite). ARTIST was a negative study because there was no statistical difference in its primary endpoint, 3‐year disease‐free survival (DFS) rate, between the two groups. A recently published update 18 confirmed the improved DFS with adjuvant chemoradiotherapy for node‐positive patients, however, there was not any OS improvement despite prolonged follow‐up interval. ARTIST‐2 trial is currently assessing the benefit of adjuvant chemoradiotherapy in node‐positive gastric cancer patients after curative resection.

CRITICS is an international, multicenter, phase 3 study that enrolled patients with resectable gastric or GEJ adenocarcinoma (stage Ib–IVa) 19. Following neodjuvant chemotherapy with ECX or EOX (epirubicin, oxaliplatin, capecitabine) and at least D1 surgery, patients were randomized to receive either the same chemotherapy or chemoradiotherapy with cisplatin and capecitabine. An almost identical 5‐year survival rate was demonstrated in the chemotherapy and the chemoradiotherapy arm (40.8% vs. 40.9%, respectively), confirming a similar degree of efficacy between the two treatment approaches (*P* = 0.99). However, 52% of patients in the chemotherapy arm and 47% in the chemoradiotherapy arm did not complete or even start the full course of either therapy assigned. These data from CRITICS emphasize the importance of preoperative treatment approach to prevent locoregional recurrence as this approach seems to be feasible and preliminary results are encouraging. For this reason, the ongoing randomized phase 2 study (CRITICS II) will compare three preoperative strategies: chemotherapy, chemoradiotherapy, and combination chemotherapy and chemoradiotherapy.

On the other hand, the ongoing international TOPGEAR trial directly compares neoadjuvant ECF versus two cycles of ECF followed by concurrent chemoradiotherapy in patients with localized resectable disease 20. In the interim safety/feasibility analysis of the trial, the proportion of patients who received all cycles of preoperative chemotherapy was 93% (ECF group) and 98% (chemoradiation group), while 65% and 53%, respectively, received all cycles of postoperative chemotherapy. The toxicity was comparable between the two arms confirming the feasibility of this approach. The results of this trial will therefore be helpful to define the sequence of multimodality treatments for localized gastric cancer worldwide.

#### Postoperative chemotherapy

Postoperative adjuvant chemotherapy with S‐1 following a D2 nodal dissection showed OS and RFS benefit in Japan 21. A second Asian study, the CLASSIC trial randomly assigned 1035 patients to receive capecitabine and oxaliplatin (CapeOx) for 6 months after gastrectomy with D2 lymphadenectomy or observation. A DFS benefit was shown in CapeOx‐treated patients (at 3 years; HR = 0.56, 95% CI: 0.44–0.72, *P* < 0.0001). Estimated 5‐year OS was 78% in the adjuvant CapeOx arm versus 69% in the observation arm 22.

In accordance with the previously analyzed studies and meta‐analyses, perioperative chemotherapy (Europe), postoperative chemoradiotherapy (United States), and postoperative chemotherapy after gastrectomy with a D2 nodal dissection (Asia) should all be considered standard of care treatment options for localized gastric cancer. Table 1 summarizes the major phase 3 trials in the localized gastric cancer setting.

**Table 1:** Major phase 3 trials in the localized gastric cancer setting

Trials	No. of patients	Treatment arms	HR for death (*P* value)	Survival Outcomes
Perioperative and preoperative chemotherapy				
Cunningham et al. 9 (MAGIC)	503	ECF →Surgery →ECF versus Surgery	0.75 (0.009)	5‐year OS: 36.3% versus 23%
Schuhmacher et al. 13(EORTC 40954)	144	CFL →Surgery (only preoperative CT) versus Surgery	0.84 (0.466)	2‐year OS: 72.7% versus 69.9%
Al‐Batran et al. 16(FLOT4‐AIO)	716	ECF/ECX→Surgery →ECF/ECX versusFLOT→Surgery →FLOT	0.77 (0.012)	3‐year OS: 48% versus 57%
Postoperative chemoradiotherapy				
Macdonald et al. 10, Smalley et al. 17(INT‐ 0116)	556	Surgery →FL/CTRT (45 Gy+FL)/FL versus Surgery	1.32 (0.0046)	3‐year OS: 50% versus 41% (OS: 36 versus 27 months)
Park et al. 18 (ARTIST)	458	Surgery →XP/XRT/XP versus Surgery →XP	1.130 (0.5272),HR for relapse: 0.740 (0.0922)	5‐year OS: 75% versus 73%,N+ pts: 3‐year DFS: 76% versus 72%
Verheij et al. 19 (CRITICS)	788	ECX or EOX → Surgery → XPRT versus ECX or EOX→ Surgery → ECX or EOX	NR (0.99)	5‐year OS: 40.9% versus 40.8%
Postoperative chemotherapy				
Sasako et al. 21 (ACTS‐GC)	1059	Surgery → S‐1 versus Surgery	0.669 (0.003)	5‐year OS: 71.7% versus 61.1%
Noh et al. 22 (CLASSIC)	1035	Surgery → XELOX versus Surgery	0.66 (0.0015),HR for relapse: 0.58 (<0.0001)	5‐year OS: 78% versus 69%,5‐year DFS: 68% versus 53%

HR, Hazard ratio; OS, Overall survival; 5‐FU, 5‐Fluorouracil; ECF, Epirubicin, Cisplatin and 5‐FU; CF, Cisplatin and 5‐FU; CFL, Cisplatin, 5‐FU and leucovorin; CT, Chemotherapy; ECX, Epirubicin, Cisplatin and Capecitabine; FLOT, Docetaxel, Oxaliplatin and 5‐FU/leucovorin; FL, 5‐FU and leucovorin; CTRT, Chemoradiotherapy; XP, Capecitabine and Cisplatin; XRT, Capecitabine and radiotherapy; DFS, Disease‐free survival; EOX, Epirubicin, Oxaliplatin and Capecitabine; XPRT, Capecitabine, Cisplatin and radiotherapy; NR, Not reported; XELOX, Capecitabine and Oxaliplatin.

### Advanced and metastatic disease

Prognosis of advanced or metastatic gastric cancer is poor with a 5‐year OS rate only 4%. Standard of care therapy for patients with advanced disease is chemotherapy. However, with the arrival of biologic targeted agents, it may be possible to select treatment based on the disease's molecular characteristics. Treatment of gastric cancer in this setting has not changed dramatically over the last decades, is primarily palliative and only two new agents have been approved (trastuzumab and ramucirumab). Multiple agents are active, but the most popular front‐line regimen contains a platinum compound plus a fluoropyrimidine (5‐FU, capecitabine and S‐1). Other active agents include taxanes, anthracyclines, irinotecan, and biologic agents such as trastuzumab for HER2‐overexpressing gastric cancers. Combinations of two or more cytotoxic drugs achieve higher response rates and according to one meta‐analysis improved survival compared to monochemotherapy 23.

#### First‐line therapy

In the first‐line setting, high‐level evidence exists for docetaxel 24, cisplatin/oxaliplatin 25 and trastuzumab 26.

In a phase 3 trial of 445 patients with metastatic disease, the addition of docetaxel to cisplatin and 5‐FU was superior in terms of response rate (37 vs. 25%; *P* = 0.01) and OS (9.2 vs. 8.6 months; *P* = 0.02) with a high rate of febrile neutropenia (30%) 24. Although the improvement in survival provided by the combination was statistically significant, it is questionable whether the less than 1 month OS benefit, especially in the context of significant toxicities, is clinically meaningful and should thus be avoided in poor performance patients.

In another randomized phase 3 trial of 1002 patients, oral capecitabine (X) was used instead of infusional 5‐FU and the non‐nephrotoxic oxaliplatin (O) as a substitute of cisplatin (EOX) in an effort to enhance on the regimen of ECF 25. EOX combination demonstrated a better toxicity profile and was at least as effective as ECF. The median survival times for the control arm of ECF, ECX, EOF and EOX arms were 9.9, 9.9, 9.3, and 11.2 months, respectively.

The third phase 3 trial randomized 305 patients in Japan to either S‐1 alone or S‐1 and cisplatin 27. The addition of cisplatin to S‐1 offered a significantly longer 2‐month OS benefit compared to S‐1 alone (13.0 vs. 11.0 months; HR for death = 0.77, 95% CI: 0.61–0.98, *P* = 0.04); thus providing evidence for the superiority of the addition of a platinum agent to a fluoropyrimidine as a reasonable treatment option in the metastatic setting. Table 2 summarizes the major phase 3 trials involving chemotherapeutic agents in the advanced/metastatic gastric cancer setting.

**Table 2:** Major phase 3 trials involving chemotherapeutic agents in the advanced/metastatic gastric cancer setting

Trials	No. of patients	Treatment arms	HR for death (*P* value)	Primary endpoint comparison (in months)
Advanced gastric cancer – first line				
Van Cutsem et al. 24 (V325 study group)	445	DCF versus CF	TTP: 1.47 (<0.001) OS: 1.29 (0.02)	TTP: 5.6 versus 3.7OS: 9.2 versus 8.6
Cunningham et al. 25	1,002	ECF versus ECX versus EOF versus EOX	0.80 (0.02)	OS: 9.9 versus 9.9 versus 9.3 versus 11.2
Koizumi et al. 27 (SPIRITS)	305	S‐1 + Cisplatin versus S‐1	0.77 (0.04)	OS: 13.0 versus 11.0
Advanced gastric cancer – Second line				
Ford et al. 30 (COUGAR ‐ 02)	168	Docetaxel + ASC versus ASC	0.67 (0.01)	OS: 5.2 versus 3.6
Thuss‐Patience et al. 31 (AIO)	40	Irinotecan + BSC versus BSC	0.48 (0.012)	OS: 4.0 versus 2.4

HR, Hazard ratio; OS, Overall survival; DCF, Docetaxel, Cisplatin and 5‐FU; CF, Cisplatin and 5‐FU; TTP, Time to progression; ECF, Epirubicin, Cisplatin and 5‐FU; ECX, Epirubicin, Cisplatin and Capecitabine; EOF, Epirubicin, Oxaliplatin and 5‐FU, EOX: Epirubicine, Oxaliplatin and Capecitabine; BSC, Best supportive care; ASC, Active symptom control.

Trastuzumab has been a success as the first biologic agent with documented clinical activity in the first line advanced and metastatic gastric and GEJ cancer setting. In the ToGA trial, 584 patients with HER2‐overexpressing tumors either by immunohistochemistry (IHC) or fluorescence in situ hybridization (FISH) were randomized to receive cisplatin plus a fluoropyrimidine with or without trastuzumab 26. The addition of targeted HER2‐therapy trastuzumab to chemotherapy added a 2.7 month OS benefit from 11.1 to 13.8 months (HR = 0.74, 95% CI: 0.60–0.91, *P* = 0.0046) in patients treated with this antibody. After extended follow‐up, there seems to be a smaller survival benefit from the addition of trastuzumab (HR = 0.80) 28, implying that the initially significant response to trastuzumab might be of short‐term. This trial led to the approval of the first combination of targeted agent to chemotherapy and has become the standard of care for patients with HER2‐overexpressing tumors.

Conclusively, the combination of a platinum compound and a fluoropyrimidine (5‐FU or capecitabine) containing chemotherapy, with the incorporation of trastuzumab for the HER2‐enriched population remains the standard of care in the first line setting. The trials addressing the value of targeted therapies, for example, EGFR and VEGF have been largely disappointing, as they did not use a biomarker‐enriched patient population, underscoring the importance of appropriate patient selection in randomized controlled trials and the use of predictive biomarkers to guide tailored therapy 29.

#### Second‐line therapy

There has been a long argument about the benefit of second‐line chemotherapy in metastatic gastric cancer, however, most recently published trials indicate a modest survival benefit when chemotherapy was compared to best supportive care (BSC) 30, 31. In the second line setting targeted HER2‐therapy with tyrosine kinase inhibitors (TKIs) has been a failure.

Ramucirumab is a fully humanized IgG1 monoclonal antibody receptor antagonist to bind the extracellular domain of VEGFR‐2, thereby blocking the binding of VEGF ligands and inhibiting receptor activation, thus inhibiting angiogenesis. In the REGARD trial, 355 patients who had progressed after first‐line chemotherapy were randomized to receive either ramucirumab or placebo 32. A marginal improvement in median OS was demonstrated in patients that received ramucirumab (5.2 months vs. 3.8 months; HR = 0.776, 95% CI: 0.603–0.998, *P* = 0.047) with an improvement in disease control rate from 23% to 49% and very low toxicity – 8% grade ≥3 hypertension. In the more recent RAINBOW trial, ramucirumab was added to weekly paclitaxel as second‐line therapy in 665 patients with metastatic gastric cancer, demonstrating a significant improvement in both PFS and OS over paclitaxel alone 33. Median OS was 9.6 and 7.4 months, respectively (HR = 0.81, 95% CI: 0.68–0.96, *P* = 0.017) with an overall good safety profile, sustaining patient quality of life with deferred symptom deterioration and functional status decline, further supporting its role in combination with chemotherapy.

#### Third‐line therapy

Apatinib is a small‐molecule multitargeted TKI with activity against VEGFR‐2, whose role was evaluated in 267, primarily Chinese patients, with heavily pretreated metastatic gastric cancer 34. This study met its primary endpoint showing significant improvement in OS and PFS. The median survival was 6.5 months for apatinib and 4.7 months for placebo (HR = 0.71, 95% CI: 0.54–0.94, *P* = 0.015) and the median PFS 2.6 months for apatinib and 1.8 months for placebo (HR = 0.44, 95% CI: 0.33–0.60, *P* < 0.001). This is the first phase 3 evidence for efficacy of a third‐line therapy in advanced gastric cancer and further supports angiogenesis inhibition as a target in this disease, thus leading to the phase 3 ANGEL study, which is investigating apatinib's efficacy and safety in the rest of the world 35.

The multikinase inhibitor regorafenib prolonged PFS versus placebo in 147 patients who had received 1 or 2 lines of prior chemotherapy for advanced gastric cancer in the phase 2 INTEGRATE trial 36. Median PFS was 2.6 months in the regorafenib plus BSC arm versus 0.9 months in the placebo arm (HR = 0.40, 95% CI: 0.28–0.59, *P* *<* 0.001). The investigators concluded that regorafenib was active and prolonged PFS in pretreated advanced gastric cancer and the phase 3 INTEGRATE II trial is ongoing 37. Table 3 summarizes the major phase 3 trials involving targeted agents in the advanced/metastatic gastric cancer setting.

**Table 3:** Major phase 3 trials involving targeted immunotherapeutic agents in the advanced/metastatic gastric cancer setting

Trials	No. of patients	Treatment arms	HR for death (*P* value)	Primary endpoint comparison (in months)
Advanced gastric cancer – first line				
Bang et al. 26 (ToGA)^a^	584	CX/CF + Trastuzumab versus CX/CF	0.74 (0.0046)	OS: 13.8 versus 11.1
Advanced gastric cancer – Second line				
Fuchs et al. 32 (REGARD)	355	Ramucirumab + BSC versus BSC	0.776 (0.0473)	OS: 5.2 versus 3.8
Wilke et al. 33 (RAINBOW)	665	Paclitaxel + Ramucirumab versus Paclitaxel	0.81 (0.017)	OS: 9.6 versus 7.4
Advanced gastric cancer – third line				
Li et al. 34 (Apatinib)	271	Apatinib + BSC versus BSC	0.71 (0.0149)	OS: 6.5 versus 4.7PFS: 2.6 versus 1.8
Kang et al. 46 (ONO‐4538‐12, ATTRACTION‐2)	493	Nivolumab versus Placebo	0.63 (<0.0001)	OS: 5.26 versus 4.14

HR, Hazard ratio; OS, Overall survival; CX, Cisplatin and Capecitabine; CF, Cisplatin and 5‐FU; PFS, Progression‐free survival; BSC, Best supportive care.

a Hazard ratio reduced to 0.8 on follow‐up analysis.

Another hot issue is the conversion therapy for gastric cancer. This has been the subject of much recent attention. It is defined as a surgical treatment that aims to achieve an R0 resection after chemotherapy for tumors that were originally unresectable or borderline resectable for oncological and/or technical reasons. Currently, there is no well‐established algorithm and whether such patients should receive eventual local therapies (such as chemoradiation and/or surgery) is still unclear. Yoshida et al. focus on the biology and heterogeneous characteristics of stage IV gastric cancer, proposing new categories of classification based on the absence (categories 1 and 2) or presence (categories 3 and 4) of macroscopically detectable peritoneal dissemination 38.

### New therapies

Immune checkpoint inhibitors have emerged in oncology as one of the most auspicious new areas of drug development. Substantial activity has been observed for these agents across a wide spectrum of hematologic malignancies and solid tumors. The EBV‐gastric cancer molecular subtype demonstrates high expression of interleukin‐12 and elevated PD‐L1 and PD‐L2 expression, indicating the potent presence of immune cells and reinforcing the use of immune checkpoint inhibitors in gastric cancer 39.

Pembrolizumab is a highly specific, humanized IgG4 monoclonal antibody that blocks the interaction between PD‐1 and its ligands PD‐L1 and PD‐L2. The safety and activity of pembrolizumab was investigated in heavily pretreated patients with gastric cancer in the phase 1b KEYNOTE‐012 trial 40. Thirty‐nine PD‐L1‐positive patients received pembrolizumab at the dose of 10 mg/kg every 2 weeks. A response was observed in 8 patients (22%, 95% CI: 10–39%) with a median duration of 40 weeks. Genomic profiling of nearly two‐thirds of cancers revealed a microsatellite‐instability high (MSI‐H) status in 17%, and two of the four patients with MSI‐H tumors responded. MSI‐H status correlates with high tumor mutational burden and patients with MSI‐H cancers (colorectal and noncolorectal) have demonstrated considerable responses to anti–PD1 therapy in a wide spectrum of solid tumors 41. It remains to be established if the same is observed in MSI‐H gastroesophageal cancers. Response in this study also correlated with an increased expression of an interferon gamma gene expression signature and an increase in mononuclear cell infiltrate score. These potential immunotherapy biomarkers remain to be studied and validated.

The strong signal of activity reported in this study provided justification for the large phase 2 KEYNOTE‐059 trial in 259 patients with advanced gastric or GEJ cancer. Preliminary analyses from cohorts 1 and 2 were presented at last ASCO. In cohort 1, pembrolizumab monotherapy showed encouraging efficacy and manageable safety after ≥2 prior lines of therapy (overall objective response rate (ORR) was 11.2% and 15.5% in 143 PD‐L1‐positive patients) 42, thus granting accelerated FDA approval to the agent for patients with PD‐L1 expressing advanced gastric cancer as third‐line option. In cohort 2, pembrolizumab plus 5‐FU and cisplatin showed manageable safety and encouraging antitumor activity as first‐line therapy (ORR was 60% and 68.8% in PD‐L1‐positive patients) 43; thus further exploration in this setting is warranted.

Nivolumab is another PD‐1 inhibitor tested in heavily pretreated patients with both PD‐L1‐positive and negative advanced gastric or GEJ cancer, having an ORR of 14% accompanied with an acceptable safety profile, according to results from the phase 1/2 CHECKMATE‐032 trial 44. PD‐L1 positivity (PD‐L1 expression above 1%) was associated with improved responses. In the recently published updated report, nivolumab ± ipilimumab resulted in durable responses and long‐term survival benefit in a group of heavily pretreated Western patients with advanced gastroesophageal cancer 45, which supports ongoing investigation and is in agreement with the clinical activity and manageable safety shown in Asian patients in the ONO‐4538‐12, ATTRACTION‐2 trial. This is the first phase 3, placebo‐controlled clinical study that evaluated nivolumab's activity and safety in 493 East Asian patients with heavily pretreated advanced gastric and GEJ cancer 46. The primary endpoint of the study was OS; median OS was 5.26 months for nivolumab versus 4.14 months for placebo (HR = 0.63, *P *< 0.0001). One‐year OS rate in the nivolumab arm was 26.2% versus 10.9% in the placebo arm. ATTRACTION‐2 is the first randomized phase 3 immuno‐oncology trial showing survival benefit in this group of difficult‐to‐treat patients; thus approval of nivolumab in Japan was granted as a promising new option for pretreated advanced gastric cancer (Table 3).

These positive results demonstrate that nivolumab has the potential to become the new standard of care for patients with heavily pretreated advanced gastroesophageal cancer and provide a strong rationale to support investigation of nivolumab ± ipilimumab and nivolumab + chemotherapy in earlier lines of treatment (phase 3 CHECKMATE‐649 trial is ongoing) 47 as well as nivolumab in combination with other immuno‐oncology agents or targeted therapies in the advanced setting. Given the rapid development of novel agents, traditional studies cannot efficiently evaluate all possible combinations. FRACTION (Fast Real‐time Assessment of Combination Therapies in Immuno‐Oncology) is an innovative promising clinical trial program with an adaptive platform design that allows for the addition of new immunotherapy combination regimens, as well as withdrawal of ineffective ones that is ongoing 48.

Currently, in the adjuvant setting, no effective standard of care is available after chemoradiotherapy followed by resection for patients with gastric and GEJ adenocarcinoma. The phase 3 CHECKMATE‐577 trial will assess nivolumab administration as an adjuvant therapy for patients who have undergone surgery 49. A novel immunotherapy agent, the first in its class, reduced disease progression by more than 50% in 246 patients when added to standard chemotherapy with EOX in patients with advanced gastric cancer, in accordance with the results from the international phase 2 FAST trial 50. The drug, IMAB362, is a chimeric IgG1 backbone antibody that targets claudin18.2 (CLDN18.2), which is a component of tight junction protein crucial for cell adhesion, integrity, and other tissue‐specific functions. Patients were required to have CLDN18.2‐positive tumors (2+/3+ intensity in ≥40% of tumor cells by IHC). Median PFS was 4.8 months with chemotherapy and 7.9 months with chemotherapy plus IMAB362 (HR = 0.47, *P* = 0.0001), whereas median OS was 8.4 months versus 13.2 months, respectively (HR = 0.51, *P* = 0.0001). The benefit was more pronounced among patients with high expression of CLDN18.2; staining in ≥70% of tumor cells 51.

Activation of costimulatory pathways, the combination of anti–PD‐1 and anti‐PD‐L1 agents with anti–CTLA‐4 (cytotoxic T‐lymphocyte–associated protein 4) agents, and overcoming other potential immunosuppressive pathways with agents such as IDO (indoleamine‐pyrrole 2,3‐dioxygenase) inhibitors, are active areas of further investigation. Efforts at applying immune checkpoint inhibitor therapy in the earlier treatment of advanced disease and moving these drugs into the adjuvant and neoadjuvant settings in gastric cancer are also moving forward. The potential for radiotherapy or other local ablative therapies to release tumor antigens may potentially further augment an immune response, as observed in the abscopal effect. This makes the application of immune checkpoint inhibitors to the chemoradiotherapy‐based treatment of gastric cancer a particular area of research interest.

## Conclusions

In conclusion, advances have been made and many more reference chemotherapy regimens are accessible for the treatment of patients with gastric cancer that have improved the mortality rates. However, much work still remains to be done by uncovering driver mutations of gastric cancer in individual patients and incorporating biologic agents. One of the most challenging and exciting field that appears promising is the potential of host's immune system, either through vaccines, antibodies, cell therapy and/or programmed cell death inhibitors that can potentially inhibit signaling pathways.

## Conflict of Interest

All authors do not have any conflict of interest.
